# Adaptive evolution and divergent expression of heat stress transcription factors in grasses

**DOI:** 10.1186/1471-2148-14-147

**Published:** 2014-06-30

**Authors:** Zefeng Yang, Yifan Wang, Yun Gao, Yong Zhou, Enying Zhang, Yunyun Hu, Yuan Yuan, Guohua Liang, Chenwu Xu

**Affiliations:** 1 Jiangsu Key Laboratory of Crop Genetics and Physiology/Co-Innovation Center for Modern Production Technology of Grain Crops, Key Laboratory of Plant Functional Genomics of the Ministry of Education, Yangzhou University, Yangzhou 225009, China

**Keywords:** Expression divergence, Grass family, Heat stress transcription factors, Orthologous gene clusters, Positive selection

## Abstract

**Background:**

Heat stress transcription factors (Hsfs) regulate gene expression in response to heat and many other environmental stresses in plants. Understanding the adaptive evolution of *Hsf* genes in the grass family will provide potentially useful information for the genetic improvement of modern crops to handle increasing global temperatures.

**Results:**

In this work, we performed a genome-wide survey of *Hsf* genes in 5 grass species, including rice, maize, sorghum, *Setaria*, and *Brachypodium*, by describing their phylogenetic relationships, adaptive evolution, and expression patterns under abiotic stresses. The *Hsf* genes in grasses were divided into 24 orthologous gene clusters (OGCs) based on phylogeneitc relationship and synteny, suggesting that 24 *Hsf* genes were present in the ancestral grass genome. However, 9 duplication and 4 gene-loss events were identified in the tested genomes. A maximum-likelihood analysis revealed the effects of positive selection in the evolution of 11 OGCs and suggested that OGCs with duplicated or lost genes were more readily influenced by positive selection than other OGCs. Further investigation revealed that positive selection acted on only one of the duplicated genes in 8 of 9 paralogous pairs, suggesting that neofunctionalization contributed to the evolution of these duplicated pairs. We also investigated the expression patterns of rice and maize *Hsf* genes under heat, salt, drought, and cold stresses. The results revealed divergent expression patterns between the duplicated genes.

**Conclusions:**

This study demonstrates that neofunctionalization by changes in expression pattern and function following gene duplication has been an important factor in the maintenance and divergence of grass *Hsf* genes.

## Background

With the rise in global temperatures and the rapid growth of the world’s population, the impact of heat stress on crop yield and quality has become increasingly significant. The genetic improvement of crops’ heat resistance through molecular manipulation has become extremely important. The expression levels of heat-shock genes increase rapidly when a plant is under conditions of heat stress, resulting in the rapid accumulation of heat-shock proteins (HSPs). HSPs function as molecular chaperones in preventing the accumulation of damaged proteins to maintain cellular homeostasis by protein refolding, stabilization, intracellular translocation and degradation [1,2].

The expression of HSPs is regulated by multiple mechanisms, mainly on a transcriptional level. Heat shock transcription factor (Hsf) is the master regulator in this process, playing critical roles in high-temperature stress responses and thermal tolerance [3]. The *Hsf* genes of animals and fungi play central roles in protecting cells from damage caused by various stress conditions, including heat, infection, inflammation, and pharmacological agents, via the activation of gene expression [4]. Like other transcription factors, Hsf proteins have a particular modular structure with a central helix-turn-helix motif in the N-terminal region, an adjacent domain with heptad hydrophobic A/B repeats involved in oligomerization, a nuclear-localization signal, and a C-terminal activation domain [5,6]. The *Hsf* genes can be divided into three structural classes: A, B and C. In the oligomerization domain, class A and C Hsf proteins possess an inserted sequence of 21 and 7 amino-acid residues, respectively, which is absent from class B Hsfs [7].

Plant *Hsf* genes have been isolated from various species. While other eukaryotes possess one to three *Hsf* genes, plants exhibit a dramatic expansion of this gene family [6]. For example, *Arabidopsis thaliana* and rice (*Oryza sativa*) have 21 and 25 *Hsf* genes [8], respectively. Class A *Hsfs* are involved in activating *Hsp* gene expression, plant development and responses to a variety of environmental stresses [4]. However, class B *Hsfs* mostly lack activator function, serving instead as repressors of gene expression [9]. The *Arabidopsis* genes *HsfA1d* and *HsfA1e* control the expression of *HsfA2*, suggesting that plant *Hsfs* also function as regulators of other *Hsf* genes [10]. In addition to heat-stress adaptation, many plant *Hsf* genes play important roles in responses to abiotic and biotic stresses, including drought, salt, cold, osmotic stress, pathogen attack, anoxia and submergence [11]. In addition to stress responses, some evidence indicates that plant *Hsfs* play potential roles in plant development. Transgenic *Arabidopsis* plants over-expressing *HsfA2* exhibit increased cell proliferation [11], while the rice gene *OsHsfC1b* is involved in ABA-mediated salt-stress tolerance, osmotic-stress response, and plant growth under non-stressed conditions [7].

The grass family, a large and nearly ubiquitous family of monocotyledonous flowering plants, constitutes the most economically important plant family in modern times, providing forage, building materials, fuel, and food. Although genome-wide surveys have identified the members of the Hsf gene family in some plant species [3,6,8], a more detailed evolutionary history of grass *Hsf* genes, including a selective pattern profile, has not been described yet. Understanding the adaptive evolution of the Hsf gene family will provide potential useful information for the genetic improvement of modern crops to tolerate increasing global temperatures. Here, we examine the phylogenetic relationships, adaptive evolution, and expression patterns under abiotic stresses of the Hsf gene family in five grass species for which genome sequences are available.

## Results

### Phylogenetic relationships of grass *Hsf* genes

**Figure 1:**
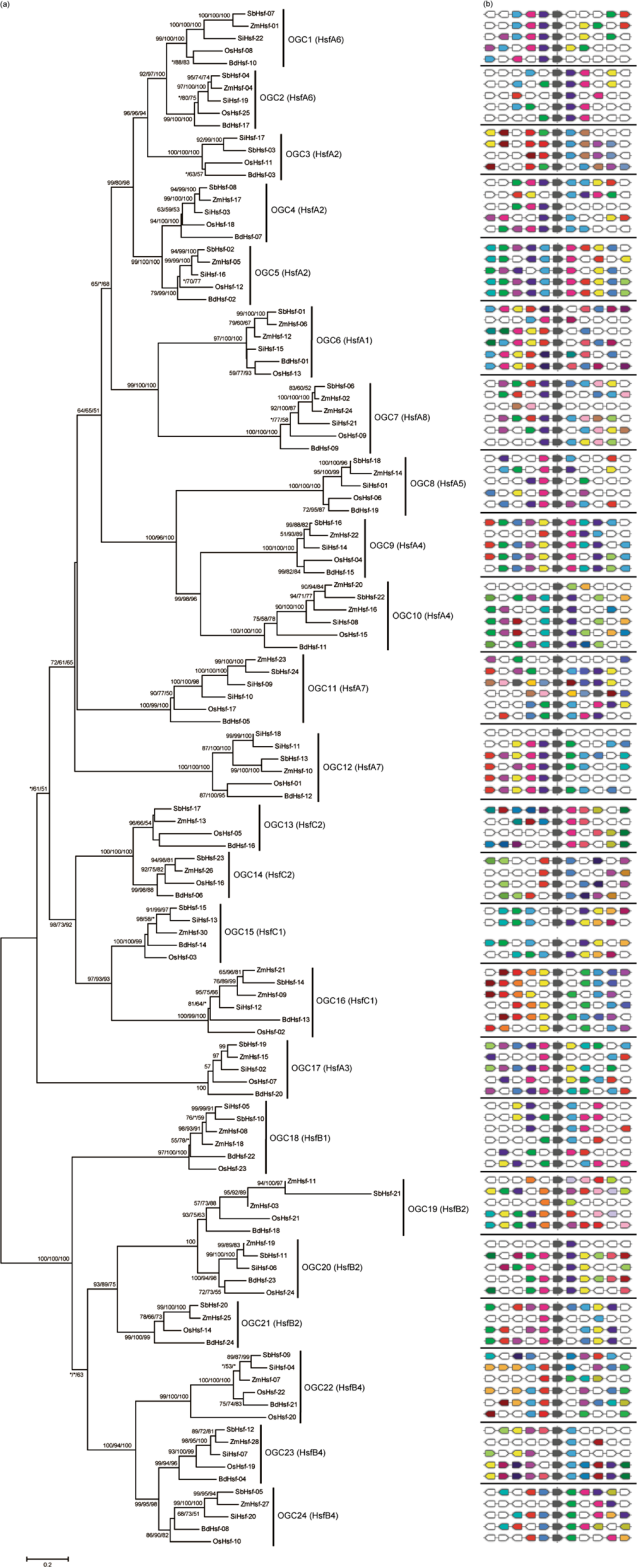
**Phylogenetic tree of grass *Hsf *genes and the synteny of each OGC. (a)** Phylogetic tree of 123 grass *Hsf* genes. The numbers above the branches indicate the maximum-likelihood, neighbor-joining and maximum parsimony bootstrap values. Asterisks indicate values smaller than 50%. The genes in the box are formed through a tandem duplication event. **(b)** Syntenic analysis for each grass *Hsf* OGC. Five protein-coding genes upstream and downstream of each *Hsf* gene are shown by depicted by colored polygons. The *Hsf* genes are shown by grey polygons. The polygons with the same color in a OGC are the homologs.

Genome-wide identification revealed 25, 24, 30, 22, and 24 *Hsf* genes in rice, *Sorghum*, maize, *Setaria* and *Brachypodium*, respectively (Additional file 1). Although the maize gene *ZmHsf-30* (GenBank entry EU954042) did not contained corresponding genomic sequence for the entire coding region, it was detected from large-scale cDNA sequencing libraries [12]. However, we noticed that the gene *ZmHsf-29* only encode a protein with 117 aa according to the annotation of maize genome, which is much smaller than other gramineous *Hsfs* (378 aa in average with a standard deviation of 72 aa)*.* We noticed that this gene might be caused by an early stop codon. Because the accuracy will tremendously decline if a gene with long regions lost is included in the phylogenetic analysis, we eliminated this gene in further analyses. To investigate the phylogenetic relationships of grass *Hsf* genes, the amino-acid sequences of the 124 *Hsf* genes were fully aligned. A combined phylogenetic tree was then reconstructed (Figure 1). In the phylogenetic tree, the grass *Hsf* genes were divided into 24 orthologous gene clusters (OGCs). In order to evaluate the rationality of classification of this result, we tested the synteny of the genes in each OGC. This analysis revealed that the *Hsf* genes within the same OGC shared the syntenic region. These results suggested that there were at least 24 *Hsf* genes in the common ancestor of these grass species and that the divergence in gene number in these species was the result of gene duplication and/or loss. The genomes of *Sorghum* and *Brachypodium* each contained 24 *Hsf* genes, with one representative in each of the 24 OGCs, suggesting that no duplication and/or loss of *Hsf* genes has occurred in these two genomes (Additional file 2). The *Setaria* genome lacked *Hsf* orthologs in 4 OGCs (OGC13, 14, 19 and 21). However, we noted one duplication event in *Setaria* in each of two OGCs (OGC11 and 12). The maize genome contained more *Hsf* genes than the other grass genomes and exhibited at least six duplication events and one loss event. The rice genome contained 25 *Hsf* genes and exhibited a single duplication event (Additional file 2). In this analysis, we also noticed that 5 *Hsf* genes possessed 2 introns in their coding regions, while all other genes contained only one. Further investigation revealed that the genes with two introns were all the members of OGC7. In this cluster, only the gene *BdHsf-09* possess only one intron, suggesting an intron loss event in this *Brachypodium* gene.

Thus, we identified 9 paralogous gene pairs formed after speciation from the common ancestor of the grass family. A search for contiguous *Hsf* genes in both the sharing region and neighboring regions revealed that only the paralogous pair *SiHsf-09/SiHsf-10* was located adjacent to another *Hsf* gene (Figure 1), suggesting that tandem duplication contributed to the formation of this paralogous pair in *Setaria*. We found that all other paralogous *Hsf* pairs, except *SiHsf-11/SiHsf-18*, were formed by large-scale gene duplication events because the flanking regions for these pairs contained highly conserved genes.

### Selective constraints on *Hsf* OGCs

To test the selective constraints on the evolution of each *Hsf*-family OGC, we compared the models M0 and M3 to detect variations in the *d*_*N*_/*d*_*S*_ ratio among codon positions within each cluster (Table 1). Overall, the average *d*_*N*_/*d*_*S*_ ratio for the 24 clusters under model M0 was 0.185 (with a standard deviation of 0.015), which was statistically smaller than 1 but greater than 0 (one-sample t-test, *p* < 0.01), illustrating that purifying selection was the predominant constraint on the evolution of the Hsf family in grasses. However, the log-likelihood differences between M3 and M0 were statistically significant for all OGCs, suggesting that the overall selective-constraint levels differed across the *Hsf* OGCs.

**Table 1:** **Detection of positive selection under site-specific models for each *Hsf*** OGC **in grasses**

**Group**	**N**	***d***_***N***_ ***d***_***/S***_**(*ω*) under M0**	**2Δ*l*, M3 *vs*. M0**	**2Δ** ***l*, M8 *vs*. M7**	**2Δ** ***l*, M8 *vs*. M8a**	**Parameter estimates under M8**	**Positively selected sites**
OGC1	5	0.123	191.391^**^	1.338	0.183	*p*_1_ = 0.055, *ω* = 1.390	NAN
						*β* (*p* = 0.241, *q* = 1.419)	
OGC2	5	0.206	83.026^**^	0.523	0.457	*p*_1_ = 0.020, *ω* = 1.975	NAN
						*β* (*p* = 0.185, *q* = 0.603)	
OGC3	4	0.110	65.437^**^	2.351	0.492	*p*_1_ = 0.028, *ω* = 2.014	NAN
						*β* (*p* = 0.537, *q* = 3.962)	
OGC4	5	0.192	76.604^**^	0.239	0.109	*p*_1_ = 0.023, *ω* = 1.370	NAN
						*β* (*p* = 0.326, *q* = 1.243)	
OGC5	5	0.198	26.238^**^	0.862	0.004	*p*_1_ = 0.085, *ω* = 1.000	NAN
						*β* (*p* = 1.347, *q* = 7.418)	
OGC6	6	0.178	84.498^**^	3.501	0.003	*p*_1_ = 0.117, *ω* = 1.027	NAN
						*β* (*p* = 0.940, *q* = 8.351)	
OGC7	6	0.337	140.949^**^	8.727^*^	5.12^*^	*p*_1_ = 0.009, *ω* = 11.123	38
						*β* (*p* = 0.190, *q* = 0.316)	
OGC8	5	0.183	97.577^**^	1.989	0.007	*p*_1_ = 0.156, *ω* = 1.000	NAN
						*β* (*p* = 1.149, *q* = 10.522)	
OGC9	5	0.235	41.245^**^	1.071	0.001	*p*_1_ = 0.151, *ω* = 1.000	NAN
						*β* (*p* = 1.903, *q* = 11.514)	
OGC10	6	0.277	314.163^**^	52.857^**^	139.556^**^	*p*_1_ = 0.079, *ω* = 4.552	20
						*β* (*p* = 0.400, *q* = 1.083)	
OGC11	6	0.168	182.994^**^	14.431^**^	4.237^*^	*p*_1_ = 0.078, *ω* = 2.082	13
						*β* (*p* = 0.602, *q* = 3.348)	
OGC12	6	0.368	77.073^**^	4.035	3.677^*^	*p*_1_ = 0.031, *ω* = 3.219	NAN
						*β* (*p* = 0.422, *q* = 0.646)	
OGC13	4	0.133	93.493^**^	8.310^*^	8.071^**^	*p*_1_ = 0.069, *ω* = 320.489	9
						*β* (*p* = 0.354, *q* = 2.050)	
OGC14	4	0.120	99.704^**^	13.741^**^	8.671^**^	*p*_1_ = 0.053, *ω* = 9.450	11
						*β* (*p* = 0.287, *q* = 2.201)	
OGC15	5	0.119	117.726^**^	21.549^**^	4.377^*^	*p*_1_ = 0.073, *ω* = 1.958	10
						*β* (*p* = 1.077, *q* = 15.385)	
OGC16	6	0.230	573.126^**^	53.223^**^	39.550^**^	*p*_1_ = 0.176, *ω* = 3.966	46
						*β* (*p* = 0.204, *q* = 0.983)	
OGC17	5	0.322	246.893^**^	27.006^**^	12.428^**^	*p*_1_ = 0.170, *ω* = 1.950	42
						*β* (*p* = 0.729, *q* = 3.471)	
OGC18	6	0.159	236.693^**^	20.455^**^	13.897^**^	*p*_1_ = 0.396, *ω* = 25.830	12
						*β* (*p* = 0.256, *q* = 1.140)	
OGC19	5	0.155	106.439^**^	3.222	1.025	*p*_1_ = 0.036, *ω* = 2.148	NAN
						*β* (*p* = 0.574, *q* = 2.971)	
OGC20	5	0.114	86.676^**^	31.524^**^	19.449^**^	*p*_1_ = 0.021, *ω* = 51.561	9
						*β* (*p* = 0.781, *q* = 6.181)	
OGC21	4	0.153	39.035^**^	6.435^*^	0.217	*p*_1_ = 0.075, *ω* = 1.257	NAN
						*β* (*p* = 2.719, *q* = 22.603)	
OGC22	6	0.093	170.968^**^	6.102^*^	1.504	*p*_1_ = 0.018, *ω* = 3.010	NAN
						*β* (*p* = 0.370, *q* = 3.227)	
OGC23	5	0.120	170.766^**^	7.271^*^	4.569^*^	*p*_1_ = 0.019, *ω* = 10.451	9
						*β* (*p* = 0.153, *q* = 0.828)	
OGC24	5	0.147	196.562^**^	11.410^**^	1.287	*p*_1_ = 0.204, *ω* = 1.236	NAN
						*β* (*p* = 3.990, *q* = 99.000)	

*Indicates significant at 0.05 level, while ** means significant at 0.01 level.

To evaluate whether positive selection facilitated the evolution of each *Hsf* OGC in grasses, we compared the models M8 and M7. This analysis indicated that 11 OGCs had undergone positive selection during the evolution of grasses because they satisfied the following criteria: (1) an estimate of *ω* > 1 under M8, (2) sites found to be under positive selection, and (3) a statistically significant likelihood ratio test (LRT). In addition, 11 OGCs were further affirmed by the LRT of the models M8 and M8a, another comparison to detect positive selection. This result suggested that positive selection was an important contributor to the evolution of at least 11 *Hsf* OGCs in grasses. Among the 11 OGCs with no gene duplication and/or loss, 4 clusters showed signals of positive selection. 5 out of 8 OGCs with gene duplications were evidently influenced by positive selection, while 2 of the 4 OGCs with gene-loss events showed evidence of positive selection. Only one OGC contained both gene-duplication and gene-loss events, and this cluster showed no signature of positive selection.

### Selective constraints on duplicated genes

**Figure 2:**
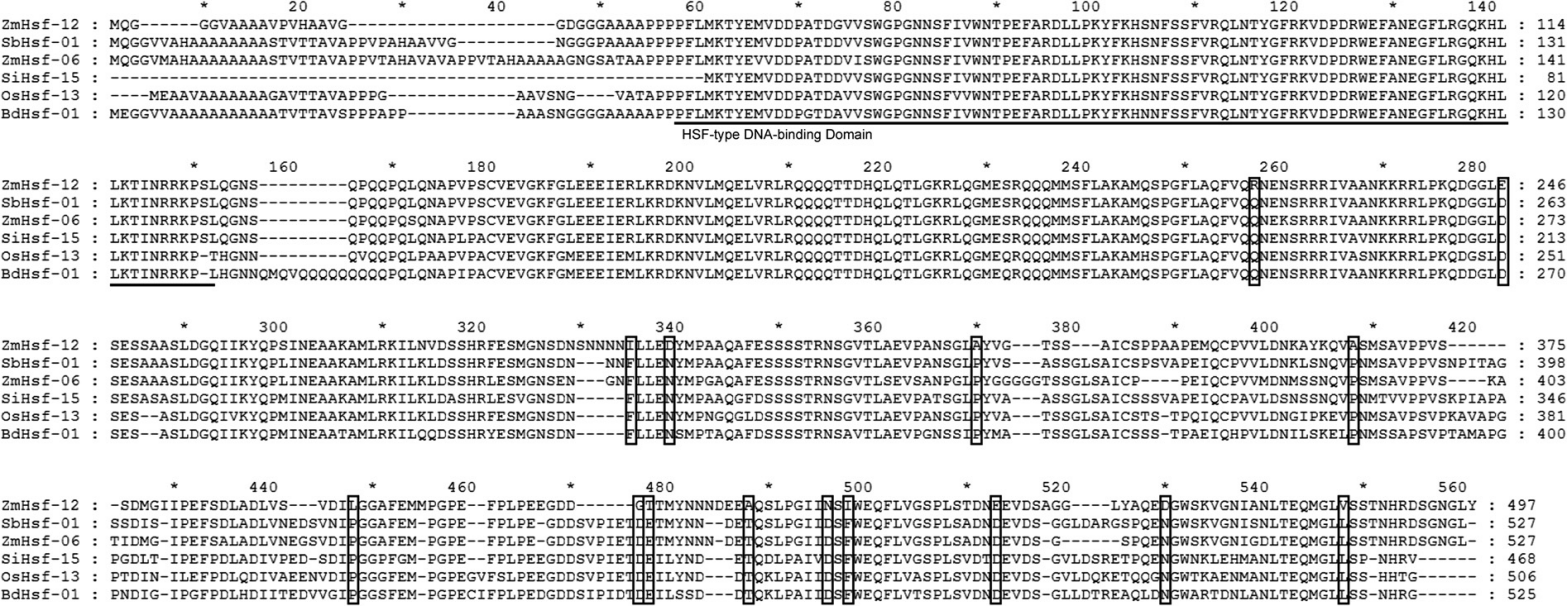
**An alignment of the Hsf protein sequences in OGC6, showing the positively selected sites in *ZmHsf-12*.** The conserved domain is indicated by underlining. The positively selected sites are indicated by deckling.

In this analysis, we also observed 9 paralogous *Hsf* gene pairs in the surveyed genomes. These paralogous gene pairs originated from duplication events after the origin of the grasses. To evaluate the selective constraints on the evolution of these duplicated genes, we used improved branch-site models [13] to examine the impact of positive selection at specific sites for each recently duplicated gene. The results revealed that only one gene in each duplicated pair was influenced by positive selection, except in the pair *SiHsf-18*/*SiHsf-11* (Additional file 3). An notable finding is that some estimated values of *d*_*N*_/*d*_*S*_ are much high or infinity under alternative model. This is the result of that some positively selected sites lack of synonymous substitution. For instance, the maize genes *ZmHsf-06* and *ZmHsf-12* were assigned to OGC6 in the phylogeny, illustrating that they constituted a duplicated gene pair that originated after the origin of the grasses. This duplicated pair formed through a large-scale gene duplication event, because other conserved genes were found in their flanking regions. In addition, both of the chromosomal regions of these two maize genes shown synteny with other genes in OGC6 (Figure 1). When we used *ZmHsf-06* as the foreground and all other genes in this OGC as the background, we found no evidence for positive selection. However, when we used *ZmHsf-12* as the foreground, the LRT revealed that positive selection contributed to the evolution of this gene (LRT = 4.992, *p* < 0.05). Because the LRT suggested the presence of positively selected sites, we implemented the Bayes empirical Bayes (BEB) method [14] to calculate posterior probabilities for each site in this gene. The BEB analysis identified 15 positively selected sites in this gene (Figure 2). Although 3 (sites 326, 477 and 478) of these sites seemed to be false positive sites that caused by InDel, all other sites located in the evolutionary conserved regions. For example, the codon 257 was found to be positively selected by BEB analysis, and the flanking amino acid sites of this codon showed evolutionary conservation among the genes of OGC6. However, the codon 257 encodes a R (arginine, Arg) in *ZmHsf-12* but a Q (glutamine, Gln) in all other genes in this OGC. Thus, these sites may be related to structural variation and may directly influence the protein function.

### Expression patterns of rice *Hsf* genes

**Figure 3:**
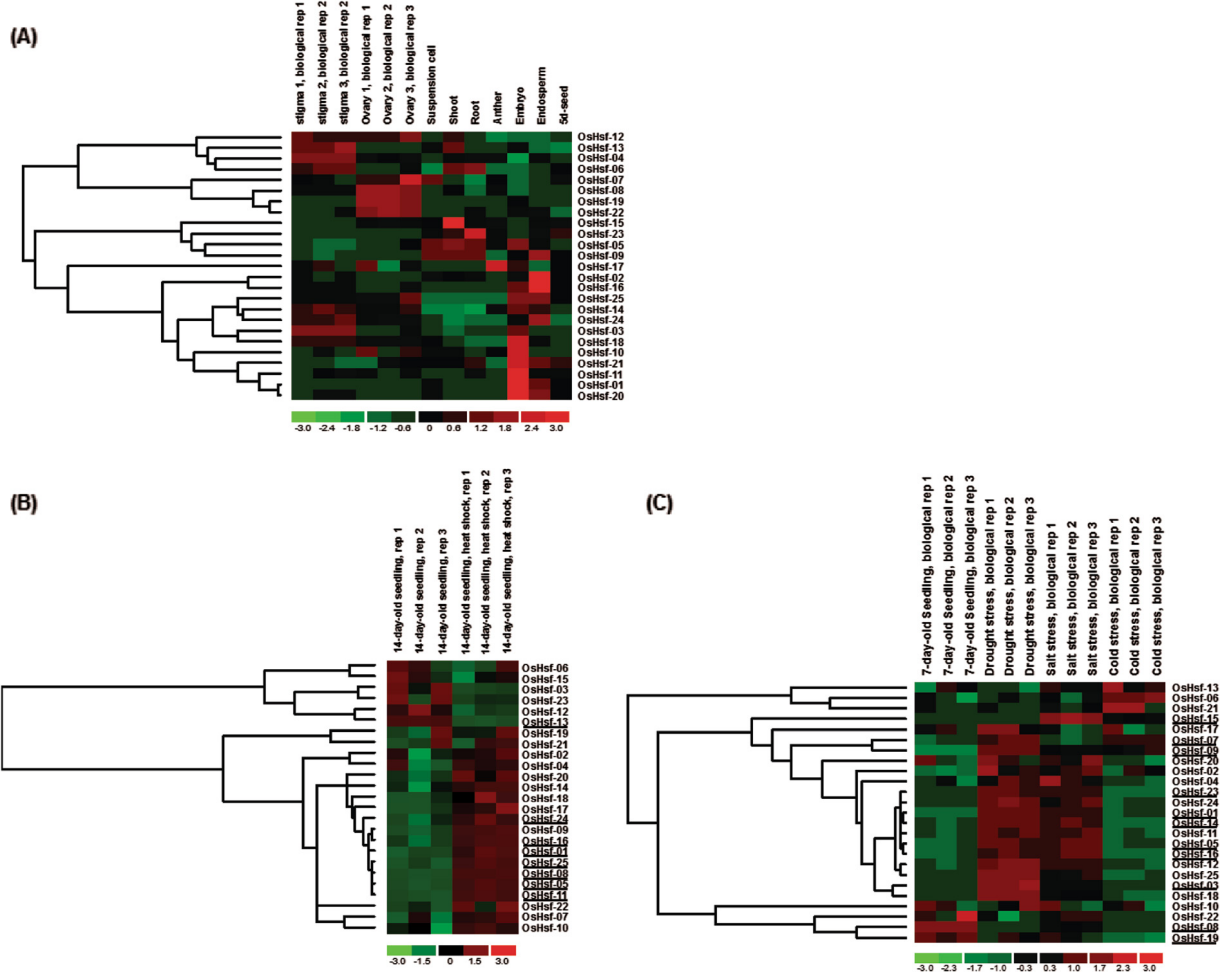
**Gene-expression patterns of rice *Hsf *genes (A) in nine tissues, (B) in response to heat shock, and (C) in response to drought, salt and cold stress.** The underlines indicate the genes shown statistically different expression levels in response to abiotic stresses compared to normal conditions.

Studying the gene-expression patterns of all members of a gene family would provide insight into their functional divergence [15]. In this analysis, we first investigated the expression patterns of rice *Hsf* genes in 9 tissues (Figure 3A). The rice *Hsf* genes were unevenly expressed in the tested tissues; furthermore, some genes clearly exhibited a tissue-specific expression pattern. For example, the gene *OsHsf-15* was mainly expressed in the shoot, while *OsHsf-02* and *OsHsf-16* were predominantly expressed in the endosperm. These results suggest that these genes play specific roles in the corresponding tissues. We also observed that the expression patterns of the one paralogous pair found in the rice genome differed strongly. The gene *OsHsf-22* was mainly expressed in the ovary, while its paralogous partner, *OsHsf-20*, was most highly expressed in the embryo, suggesting that functional divergence occurred between these genes after duplication.

We also analyzed the expression profiles of rice *Hsf* genes under control and heat-shock conditions. The rice *Hsf* genes exhibited two distinct expression patterns. Six rice *Hsf* genes were suppressed under heat-shock conditions compared to the control, while the remaining 19 genes were induced by heat-shock conditions (Figure 3B). When we used the program SAM to identify the genes with significant changes in expression between control and heat-shock conditions, we found that only 1 gene was significantly down-regulated by heat shock, while 7 genes were up-regulated by two-fold or greater (Additional file 4), respectively. We also observed that most of the rice *Hsf* genes were up-regulated under a variety of stresses, such as drought, salt, and cold. Among the 25 rice *Hsf* genes, 5, 6 and 4 genes were statistically up-regulated by drought, salt and cold, respectively (Figure 3C, Additional file 5). In addition, 2 genes were down-regulated by cold treatment.

### Expression patterns of maize *Hsf* genes in response to abiotic stresses

**Figure 4:**
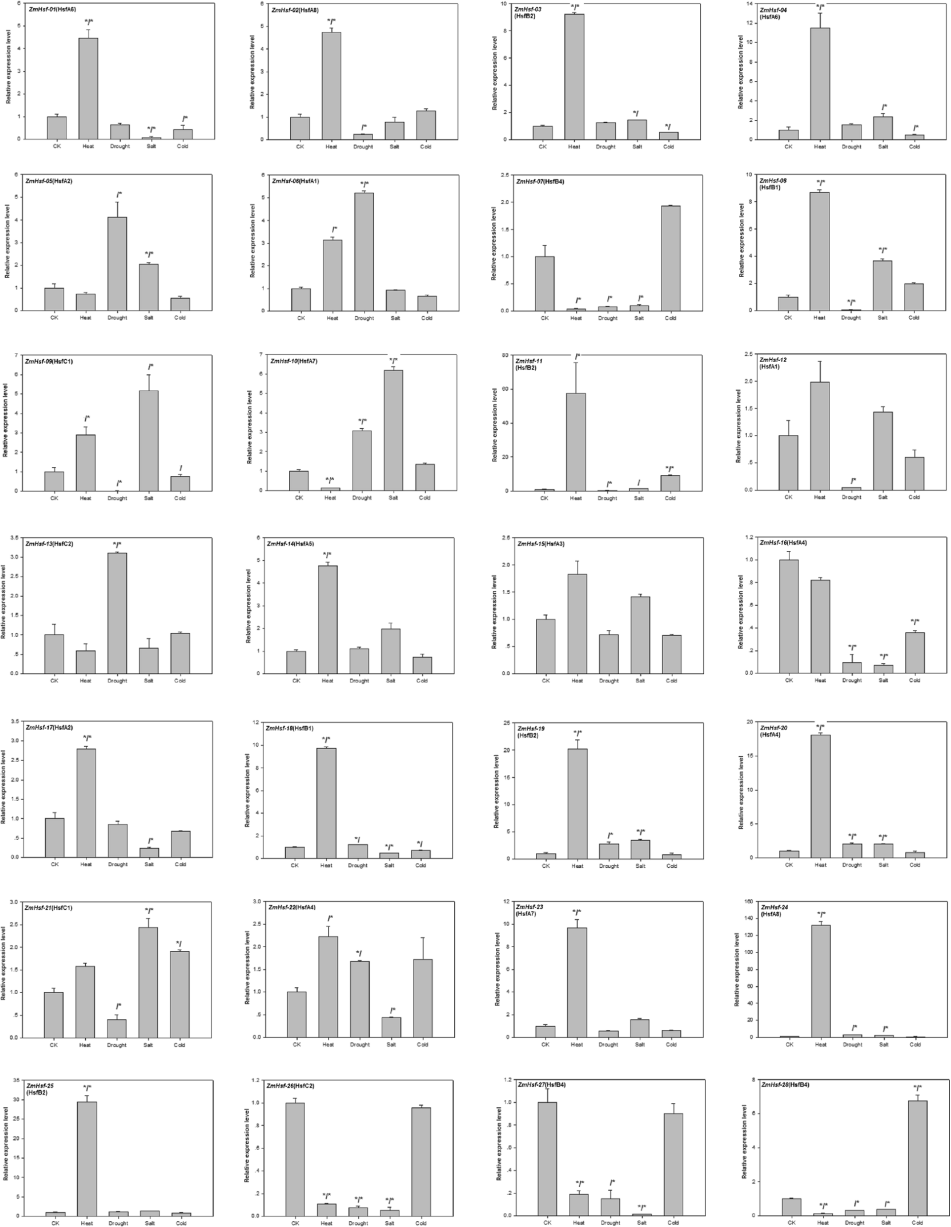
**Relative gene expression of *ZmHsfs *in response to heat, drought, salt, and cold treatments, analyzed by qRT-PCR.** An asterisk before the slash indicates a statistically significant difference in expression compared to CK (*t*-test), while an asterisk after the slash indicates a two-fold or greater difference in expression.

Because expression patterns can provide important clues to the functional divergence of paralogous gene pairs, we further investigated the expression of maize *Hsf* genes in response to abiotic stress. In this analysis, we detected the expression levels of 28 maize *Hsf* genes under heat, drought, cold and salt stresses by real-time PCR (qPCR) analysis. When we used *t*-tests to identify significant differences in expression, we found that 13, 5, 6 and 3 genes were up-regulated at the 0.05 level by heat, drought, salt and cold, respectively (Figure 4). In addition, 4, 4, 5 and 3 genes were down-regulated under heat, drought, salt, and cold, respectively. When we used the criterion of a two-fold or greater change in expression, we found that 18, 7, 8 and 2 maize *Hsf* genes were up-regulated by the same stresses, while 5, 10, 10 and 3 were down-regulated.

The maize genome contained 6 recently duplicated *Hsf* pairs. We further investigated the expression patterns of these paralogous pairs and found that each gene showed a differential expression pattern compared to its paralogous partner. For example, the gene *ZmHsf-16* was strongly down-regulated by drought, while its paralog (*ZmHsf-20*) was strongly up-regulated by drought. These results indicate functional divergence between the members of maize *Hsf* duplicated pairs.

## Discussion

Gene duplication is a major mechanism through which new genetic material is generated during evolution. Among gene-duplication mechanisms, whole genome duplication (WGD) is the dominant mechanism for gene-family proliferation in plants because most plants are diploidized polyploids and retain numerous duplicated chromosomal blocks within their genomes [16,17]. For example, the rice genome shows evidence for two rounds of ancient polyploidy events: one before the divergence of cereals and one before the split between monocots and dicots [18]. In addition, it is generally believed that maize arose as a tetraploid [19,20]. Here, we found 9 paralogous pairs of *Hsf* genes in 3 grass species. These paralogous pairs were formed after the divergence of the grasses, and at least 7 pairs were formed by large-scale gene duplication events. Among the surveyed grass genomes, maize possesses the largest number of *Hsf* genes, although its genome also shows gene-loss events. It is easier to infer that most recent duplicated maize *Hsf* genes formed through WGD because of the tetraploid process. The rice paralogous pair *OsHsf-20/22* were also found to be formed through a large-scale duplication evnet. However, we also noticed that the rice chromosomes 8 and 9 formed through a whole-genome duplication event before the split of cereals [18,21]. It was also suggested that reciprocal gene loss following a WGD can contribute to reproductive isolation through divergent resolution of duplicate copies, foreshadowing the diversification of species [18]. Thus, the most acceptable duplicated pattern for the pair of *OsHsf20/22* is that these two genes formed through a WGD event before the split of grasses, and have lost one partner in other cereal genomes.

Abiotic stresses have significant impacts on plants over the long term. Plants have successfully evolved enzymes and regulatory mechanisms to adapt to their environments, including abiotic stresses [22]. However, global environments have changed tremendously during the long period of plant evolution. To adapt successfully, a plant must overcome deleterious new conditions without creating different but equally dangerous alterations in its ongoing successful metabolic relationship with its environment [23]. Thus, stress-response genes are readily influenced by adaptive evolution. Adaptive evolution results from the spread of advantageous mutations through positive selection, which is thought to be the most important mechanism to generate new gene functions [24]. Genes carrying signatures of selection may be involved in adaptation and functional innovation. The *d*_*N*_/*d*_*S*_ ratio measures the selective pressure on amino-acid substitutions. A *d*_*N*_/*d*_*S*_ ratio greater than 1 suggests positive selection, while a ratio less than 1 suggests purifying selection [25]. The members of the Hsf family encode key regulators of physiological responses to heat and other abiotic stresses [5,6,8]. In an explicit evolutionary analysis of gains/losses, we show that the ancestor of grasses had 24 *Hsf* genes. As a result of evolution, modern grass species contain 24 *Hsf* OGCs. Positive selection has affected 11 of these OGCs during the evolution of the grass family. This result suggests that positive selection has played important roles in the evolution of the Hsf family in grasses. Interestingly, we observed that the OGCs with gene duplications and/or losses tended to show stronger evidence of positive selection. Because positive selection is believed to indicate the evolution of new functions, OGCs that contain only one member in each genome may have fewer chances to acquire new functions [26]. However, gene duplication provides new genetic material to evolve new functions through positive selection, possibly explaining why most OGCs that contained duplicated genes were influenced by positive selection. The members of the OGCs that contained gene losses may not play housekeeping roles in grasses because the species that lack these genes have survived over long evolutionary periods. Thus, a possible explanation for the positive selection found in these OGCs is that these genes are subject to the relaxed constraints of purifying selection, and positive selection has helped them to fit the beneficial variants.

Ortholog refer to the homologous genes where a gene is found in two different species, but the origin of the gene is a common ancestor. If a gene is duplicated in a species, the resulting duplicated genes are paralogs of each other. Orthologs generally retain the same function over the course of evolution. However, paralogs commonly evolve new functions, although these functions may be related to the original function, especially for those formed through lineage-specific duplication events [27-29]. During the gene-duplication process, paralogs commonly undergo a division of labor by retaining different parts (subfunctions) of their original ancestral function. This process is known as subfunctionalization. However, a gene may instead acquire a new function after gene duplication. This process is known as neofunctionalization [30]. Gene duplication results in an additional copy that is free from selective pressure. If the duplicated pair does not undergo subfunctionalization, the additional copy may be lost due to the accumulation of natural mutations unless it acquires new functions through positive selection [31]. Thus, the signal of positive selection indicates neofunctionalization for one of the duplicated genes [32]. In this analysis, we tested for signals of positive selection in 9 duplicated pairs of *Hsf* genes in the surveyed grass genomes. We also tested the expression levels of 7 duplicated gene pairs in rice and maize and found that all of these duplicated pairs showed divergent expression patterns. This result suggests that subfunctionalization and/or neofunctionalization has occurred after duplication in response to different stresses. Our results also indicate that positive selection has acted on only one paralog within 8 pairs, while the other gene within each pair shows no evidence of positive selection. Thus, one gene in each pair likely retained the original function, while the other gene may have gained new functions through positive selection. The signatures of positive selection and divergent expression suggest that neofunctionalization has contributed to the evolution of duplicated *Hsf* genes. Our findings provide a novel reference for cloning the most promising candidate genes from the Hsf gene family for further functional detection.

## Conclusions

Based on the phylogeny and syntenic information, the *Hsf* genes in five gramineous genomes were divided into 24 orthologous gene clusters (OGCs), suggesting that there were at least 24 *Hsf* genes in the common ancestor of these grass species and that the divergence in gene number in these species was the result of gene duplication and/or loss. In addition, 9 duplication and 5 gene-loss events were identified in the tested genomes. Among the 11 OGCs with no gene duplication and/or loss, 4 clusters showed signals of positive selection. However, 7 out of 13 OGCs with gene duplication and/or loss were evidently influenced by positive selection, suggesting that OGCs with duplicated or lost genes were more readily influenced by positive selection than other OGCs. When we used the improved branch-site model to test adaptive evolution for the recently duplicated *Hsf* genes, the results revealed that positive selection acted on only one of the duplicated genes in 8 of 9 paralogous pairs. Furth more, we also investigated the expression patterns of rice and maize *Hsf* genes under heat, salt, drought, and cold stresses, and the results revealed divergent expression patterns between the duplicated genes. This study demonstrates that neofunctionalization by changes in expression pattern and function following gene duplication has been an important factor in the maintenance and divergence of grass *Hsf* genes.

## Methods

### Identification of *Hsf* genes in grasses

To identify the members of the Hsf gene family in rice (*Oryza sativa*), maize (*Zea mays*), *Sorghum bicolor*, *Setaria italica* and *Brachypodium distachyon*, *Hsf* gene sequences from *Arabidopsis*[8] were retrieved from the TAIR database [33] and used as queries to perform repetitive BLAST searches against the Phytozome database v9.1 [34]. BLAST searches were also performed against the NCBI nucleic-acid sequence data repositories. All protein sequences derived from the BLAST searches were examined using domain-analysis programs, including Pfam [35] and SMART [36], with the default cut-off parameters.

### Multiple sequence alignment and phylogenetic tree reconstruction

Multiple sequence alignment of Hsf proteins was performed using the program Clustal X [37] with the default parameters. Phylogenetic analyses were performed using neighbor joining (NJ), maximum parsimony (MP) in the program MEGA version 5.1 [38] and using maximum likelihood (ML) in the program PhyML version 3.0 [39]. Modelgenerator [40] analysis revealed that a JTT substitution matrix was the most appropriate parameter for the alignment dataset. The ML phylogenetic analyses used the following parameters: JTT model, estimated proportion of invariable sites, 4 rate categories, estimated gamma-distribution parameter, and BIONJ-optimized starting tree. The JTT model was also used for the construction of NJ trees. A total of 100 non-parametric bootstrap samplings were performed to estimate the support level for each internal branch in the ML, NJ and MP trees. The branch lengths and topologies of all phylogenies were calculated using PhyML. The phylogenetic trees were visualized using the explorer program in MEGA.

### Synteny analysis

The synteny relationships of *Hsf* genes in one OGC were analyzed by reciprocal BLASTP. 5 protein-coding genes upstream and downstream of each *Hsf* gene were obtained from the Phytozome database [34]. The genes flanking one *Hsf* gene were used to match the genes flanking other *Hsf* gene in the same OGC using reciprocal BLASTP. Therefore, we considered *Hsf* genes in the same OGC to share syntenic region if they resided within a region of other conserved protein-coding genes. The detection of conserved protein-coding gene used the tool of BLASTP with the E-value ≤ 1*E* - 10.

### Detection of positive selection

The program PAL2NAL [41] was used to convert the protein sequence alignment into the corresponding codon-based nucleotide alignment, which was input into the *codeml* program in PAML [42]. Using the program *codeml*, we detected variation in the *ω* parameter among sites by employing likelihood-ratio tests (LRTs) for M0 *vs.* M3 and M7 *vs.* M8. The LRT for the M0 *vs*. M3 comparison was used to test the heterogeneity in *ω* between codon positions, where M0 model assumes one *ω* among all sites and M8 model uses an unconstrained discrete distribution of *ω* with a set number of classes. The M7 *vs.* M8 comparison was used to detect the role of positive selection. M7 is the null model assumes a beta distribution of *ω* values between *ω* = 0 and *ω* = 1 among the sites, while the alternative model M8 adds a free parameter to the null model and allows positive selection to occur. In each LRT, twice the difference of the log likelihood of the two models was compared to the chi-squared (*χ*^2^) statistic, with the degrees of freedom (DFs) being equal to the difference in the number of parameters. In our analyses, the DFs were 4 for the M0 *vs*. M3 test and 2 for the M7 *vs*. M8 test [43]. Additionally, to stringently test for evidence of positive selection and to remove the potential identification of relaxed purifying selection, we conducted a comparison of M8 model (where a single class of sites is allowed with *ω* > 1) to M8a, which is specified using *ω* = 1 [44].

An improved branch-site model [13] was also used to detect the impact of positive selection upon one gene in each duplicated pair. For this analysis, we compared the null hypothesis (*ω* fixed to 1) with the alternative hypothesis (free *ω*) to test whether positive selection acted upon the genes in duplicated pairs. In this analysis, each gene in a duplicated pair was used as the foreground, while the other genes in the same OGC were used as the background. The BEB procedure [14] in *codeml* was used to calculate the posterior probability that each site in the foreground branch was subject to positive selection.

### Microarray data analysis

The microarray data publicly available from the GEO database under the series accession numbers GSE7951 (expression profiling of 9 rice tissues), GSE6901 (expression data for heat, cold and salt treatments), and GSE14275 (expression data for heat-shock conditions) were used in an expression analysis of rice *Hsf* genes. The program dChip version 2010 [45] was used to perform the cluster analysis and to display the expression patterns of rice *Hsf* genes based on the microarray data. Gene-expression values were compared using the program Significance Analysis of Microarrays (SAM) [46] in Microsoft Excel based on the criterion of more than two-fold change. In this analysis, SAM two-class unpaired analysis was used to calculate *p*-values, *q*-values and fold changes in expression levels.

### Plant materials and stress treatment

The maize inbred line Huangzaosi was used to check the gene-expression levels of maize *Hsf* genes. The maize plants were grown until the four-leaf stage under natural light and environmental conditions in soil-filled pots that were watered every 2 d. To induce drought stress, watering was stopped for each pot for 6 d. To induce heat stress, the pots were placed in an incubator at 42°C. To induce cold stress, the pots were placed in an incubator at 4°C. To induce salt stress, the pots were watered with 200 mM NaCl in water. The leaves were sampled after 4 h of heat, salt or cold treatment.

### RNA isolation and quantitative real-Time PCR (qrt-PCR) analyses

Total RNA was extracted from Huangzaosi maize plants subjected to four stress treatments using an RNAsimple Total RNA Kit (Tiangen). The RNA was stored at -72°C and reverse-transcribed into cDNA using PrimeScript RT Master Mix Perfect Real Time (TaKaRa). Real-time quantitative PCR was performed using 2 μl of cDNA in a 25-μl reaction volume with SYBR Premix Ex Taq (TaKaRa), utilizing the Applied Biosystems 7500 Real-Time PCR System. Gene-specific primers were designed using the program Primer 5.0 (Additional file 5). The *Zea mays Actin* gene was used as an internal reference for all qRT–PCR analyses. Each treatment was repeated three times independently. The reaction profile consisted of an initial incubation at 50°C for 2 min and 95°C for 5 min, followed by 40 cycles at 95°C for 30 s, 54°C for 30 s and 72°C for 40 s. The relative quantification of *Hsfs* transcript levels was achieved using the comparative *C*_*T*_ method (also known as the $2^{-\Delta \Delta C_{T}}$ method) [47]. The independent-samples *t* test was employed to compare the significant difference of all stress treatments against their controls using SAS v9.1.3 (SAS Institute Inc., USA). In this analysis, a total of 112 independent comparisons were performed, and the experiment-wide significance level were set to $\bar{\alpha }=0.05$. According to multiple testing of Šidák correction, the significance level for per comparison was defined as $\alpha =1-{\left(1-\bar{\alpha }\right)}^{1/112}=4.58E-4$. Thus, if the *P* value < *α* for an independent-samples *t* test, the significant difference between stress treatment and its control is suggested.

## Availability of supporting data

The data sets supporting the results of this article are included within the article and its additional files. Alignment and Phylogenetic tree which support the findings presented in this research article are available online in the Dryad Digital Repository (doi:10.5061/dryad.11243) [48].

## Competing interests

The authors declare that they have no competing interests.

## Authors’ contributions

ZY, YW and CX designed the study and conceived the experiments. ZY, YW and EZ characterized the sequences and carried out most of the experiments. YW, YG, YZ and YY participated expression analyses. ZY and YG performed evolutionary analyses. ZY and GL analyzed data. ZY and CX drafted the manuscript. All authors read and approved the final manuscript.

## Supplementary Material

Additional file 1 Summary of 125 Hsf genes identified in 5 grass genomes. Click here for file

Additional file 2 Distribution of gramineous Hsf genes in 24 OGCs. Click here for file

Additional file 3 Detection of positive selection in duplicated Hsf pairs in grasses using branch-site models. Click here for file

Additional file 4 Rice Hsf genes show statistically different expression levels in response to heat, drought, salt, and cold treatments compared to normal conditions. Click here for file

Additional file 5 Primer sequences used for qRT-PCR amplification. Click here for file
