# Supplementary material for: Adaptive evolution and divergent expression of heat stress transcription factors in grasses
Source: BMC Evol Biol. 2014 Jun 30;14:147. doi: 10.1186/1471-2148-14-147 (PMC4094458; doi:10.1186/1471-2148-14-147)
Supplement: Additional file 2 — Distribution of gramineous Hsf genes in 24 OGCs. [file 1471-2148-14-147-S2.doc]

**Additional file 2. Distribution of gramineous *Hsf* genes in 24 OGCs**

| OGC | Classification | *Zea mays* | *Oryza sativa* | *Sorghum bicolor* | *Setaria italica* | *Brachypodium distachyon* |
| --- | --- | --- | --- | --- | --- | --- |
| OGC1 | HsfA6 | *ZmHsf-01* | *OsHsf-08* | *SbHsf-07* | *SiHsf-22* | *BdHsf-10* |
| OGC2 | HsfA6 | *ZmHsf-04* | *OsHsf-25* | *SbHsf-04* | *SiHsf-19* | *BdHsf-17* |
| OGC3 | HsfA2 |  | *OsHsf-11* | *SbHsf-03* | *SiHsf-17* | *BdHsf-03* |
| OGC4 | HsfA2 | *ZmHsf-17* | *OsHsf-18* | *SbHsf-08* | *SiHsf-03* | *BdHsf-07* |
| OGC5 | HsfA2 | *ZmHsf-05* | *OsHsf-12* | *SbHsf-02* | *SiHsf-16* | *BdHsf-02* |
| OGC6 | HsfA1 | *ZmHsf-06; ZmHsf-12* | *OsHsf-13* | *SbHsf-01* | *SiHsf-15* | *BdHsf-01* |
| OGC7 | HsfA8 | *ZmHsf-02; ZmHsf-24* | *OsHsf-09* | *SbHsf-06* | *SiHsf-21* | *BdHsf-09* |
| OGC8 | HsfA5 | *ZmHsf-14* | *OsHsf-06* | *SbHsf-18* | *SiHsf-01* | *BdHsf-19* |
| OGC9 | HsfA4 | *ZmHsf-22* | *OsHsf-04* | *SbHsf-16* | *SiHsf-14* | *BdHsf-15* |
| OGC10 | HsfA4 | *ZmHsf-20; ZmHsf-16* | *OsHsf-15* | *SbHsf-22* | *SiHsf-08* | *BdHsf-11* |
| OGC11 | HsfA7 | *ZmHsf-23* | *OsHsf-17* | *SbHsf-24* | *SiHsf-09; SiHsf-10* | *BdHsf-05* |
| OGC12 | HsfA7 | *ZmHsf-10* | *OsHsf-01* | *SbHsf-13* | *SiHsf-18; SiHsf-11* | *BdHsf-12* |
| OGC13 | HsfC2 | *ZmHsf-13* | *OsHsf-05* | *SbHsf-17* |  | *BdHsf-16* |
| OGC14 | HsfC2 | *ZmHsf-26* | *OsHsf-16* | *SbHsf-23* |  | *BdHsf-06* |
| OGC15 | HsfC1 | *ZmHsf-30* | *OsHsf-03* | *SbHsf-15* | *SiHsf-13* | *BdHsf-14* |
| OGC16 | HsfC1 | *ZmHsf-21; ZmHsf-09* | *OsHsf-02* | *SbHsf-14* | *SiHsf-12* | *BdHsf-13* |
| OGC17 | HsfA3 | *ZmHsf-15* | *OsHsf-07* | *SbHsf-19* | *SiHsf-02* | *BdHsf-20* |
| OGC18 | HsfB1 | *ZmHsf-08; ZmHsf-18* | *OsHsf-23* | *SbHsf-10* | *SiHsf-05* | *BdHsf-22* |
| OGC19 | HsfB2 | *ZmHsf-11; ZmHsf-03* | *OsHsf-21* | *SbHsf-21* |  | *BdHsf-18* |
| OGC20 | HsfB2 | *ZmHsf-19* | *OsHsf-24* | *SbHsf-11* | *SiHsf-06* | *BdHsf-23* |
| OGC21 | HsfB2 | *ZmHsf-25* | *OsHsf-14* | *SbHsf-20* |  | *BdHsf-24* |
| OGC22 | HsfB4 | *ZmHsf-07* | *OsHsf-22; OsHsf-20* | *SbHsf-09* | *SiHsf-04* | *BdHsf-21* |
| OGC23 | HsfB4 | *ZmHsf-28* | *OsHsf-19* | *SbHsf-12* | *SiHsf-07* | *BdHsf-04* |
| OGC24 | HsfB4 | *ZmHsf-27* | *OsHsf-10* | *SbHsf-05* | *SiHsf-20* | *BdHsf-08* |
