# Supplementary material for: Adaptive evolution and divergent expression of heat stress transcription factors in grasses
Source: BMC Evol Biol. 2014 Jun 30;14:147. doi: 10.1186/1471-2148-14-147 (PMC4094458; doi:10.1186/1471-2148-14-147)
Supplement: Additional file 3 — Detection of positive selection in duplicated Hsf pairs in grasses using branch-site models. [file 1471-2148-14-147-S3.docx]

**Additional file 3. Detection of positive selection in duplicated *Hsf* pairs in grasses using branch-site models.**

| **Foreground** | **Background** | **Model** | **$\ln L$** | **Parameters** | **Positively selected sites** |
| --- | --- | --- | --- | --- | --- |
| *ZmHsf-12* | OGC6 | Null | -4101.577 | $p_{0}=\text{ 0.703},p_{1}=0.114,p_{2a}=0.157,p_{2b}=0.025$  Background: $\omega_{0}=0.069,\omega_{1}=\text{1.000,}\omega_{2a}=0.069,\omega_{2b}=1.000$  Foreground: $\omega_{0}=0.069,\omega_{1}=\text{1.000,}\omega_{2a}=1.000,\omega_{2b}=1.000$ | - |
|  |  | Alternative | -4099.081^*^ | $p_{0}=0.795,p_{1}=0.130,p_{2a}=0.065,p_{2b}=0.011$  Background: $\omega_{0}=0.070,\omega_{1}=\text{1.000,}\omega_{2a}=0.070,\omega_{2b}=1.000$  Foreground: $\omega_{0}=0.070,\omega_{1}=\text{1.000,}\omega_{2a}=2.704,\omega_{2b}=2.704$ | 15, 1^*^ |
| *ZmHsf-06* | OGC6 | Null | -4105.785 | $p_{0}=\text{ 0.772},p_{1}=0.135,p_{2a}=0.079,p_{2b}=0.014$  Background: $\omega_{0}=0.073,\omega_{1}=\text{1.000,}\omega_{2a}=0.073,\omega_{2b}=1.000$  Foreground: $\omega_{0}=0.073,\omega_{1}=\text{1.000,}\omega_{2a}=1.000,\omega_{2b}=1.000$ | - |
|  |  | Alternative | -4105.785 | $p_{0}=0.773,p_{1}=0.135,p_{2a}=0.079,p_{2b}=0.014$  Background: $\omega_{0}=0.073,\omega_{1}=\text{1.000,}\omega_{2a}=0.073,\omega_{2b}=1.000$  Foreground: $\omega_{0}=0.073,\omega_{1}=\text{1.000,}\omega_{2a}=1.000,\omega_{2b}=1.000$ | NAN |
| *ZmHsf-24* | OGC7 | Null | -4046.429 | $p_{0}=\text{ 0.586},p_{1}=0.306,p_{2a}=0.071,p_{2b}=\text{ 0.037}$  Background: $\omega_{0}=0.069,\omega_{1}=\text{1.000,}\omega_{2a}=0.069,\omega_{2b}=1.000$  Foreground: $\omega_{0}=0.069,\omega_{1}=\text{1.000,}\omega_{2a}=1.000,\omega_{2b}=1.000$ | - |
|  |  | Alternative | -4043.187^*^ | $p_{0}=0.653,p_{1}=0.342,p_{2a}=0.003,p_{2b}=0.002$  Background: $\omega_{0}=0.072,\omega_{1}=\text{1.000,}\omega_{2a}=0.072,\omega_{2b}=1.000$  Foreground: $\omega_{0}=0.072,\omega_{1}=\text{1.000,}\omega_{2a}=271.651,\omega_{2b}=271.651$ | 3, 1^*^ |
| *ZmHsf-02* | OGC7 | Null | -4046.860 | $p_{0}=\text{ 0.587},p_{1}=0.307,p_{2a}=0.069,p_{2b}=\text{ 0.036}$  Background: $\omega_{0}=0.071,\omega_{1}=\text{1.000,}\omega_{2a}=0.071,\omega_{2b}=1.000$  Foreground: $\omega_{0}=0.071,\omega_{1}=\text{1.000,}\omega_{2a}=1.000,\omega_{2b}=1.000$ | - |
|  |  | Alternative | -4046.860 | $p_{0}=0.587,p_{1}=0.307,p_{2a}=0.070,p_{2b}=0.036$  Background: $\omega_{0}=0.071,\omega_{1}=\text{1.000,}\omega_{2a}=0.071,\omega_{2b}=1.000$  Foreground: $\omega_{0}=0.071,\omega_{1}=\text{1.000,}\omega_{2a}=1.000,\omega_{2b}=1.000$ | NAN |
| *ZmHsf-20* | OGC10 | Null | -4392.712 | $p_{0}=\text{ 0.700},p_{1}=0.300,p_{2a}=0.000,p_{2b}=\text{ 0.000}$  Background: $\omega_{0}=0.082,\omega_{1}=\text{1.000,}\omega_{2a}=0.082,\omega_{2b}=1.000$  Foreground: $\omega_{0}=0.082,\omega_{1}=\text{1.000,}\omega_{2a}=1.000,\omega_{2b}=1.000$ | - |
|  |  | Alternative | -4392.712 | $p_{0}=0.700,p_{1}=0.300,p_{2a}=0.000,p_{2b}=0.000$  Background: $\omega_{0}=0.082,\omega_{1}=\text{1.000,}\omega_{2a}=0.082,\omega_{2b}=1.000$  Foreground: $\omega_{0}=0.082,\omega_{1}=\text{1.000,}\omega_{2a}=1.000,\omega_{2b}=1.000$ | NAN |
| *ZmHsf-16* | OGC10 | Null | -4386.216 | $p_{0}=\text{ 0.517},p_{1}=0.229,p_{2a}=0.176,p_{2b}=\text{ 0.078}$  Background: $\omega_{0}=0.054,\omega_{1}=\text{1.000,}\omega_{2a}=0.054,\omega_{2b}=1.000$  Foreground: $\omega_{0}=0.054,\omega_{1}=\text{1.000,}\omega_{2a}=1.000,\omega_{2b}=1.000$ | - |
|  |  | Alternative | -4383.504^*^ | $p_{0}=0.600,p_{1}=0.269,p_{2a}=0.091,p_{2b}=0.041$  Background: $\omega_{0}=0.057,\omega_{1}=\text{1.000,}\omega_{2a}=0.057,\omega_{2b}=1.000$  Foreground: $\omega_{0}=0.057,\omega_{1}=\text{1.000,}\omega_{2a}=7.402,\omega_{2b}=7.402$ | 38, 2^*^ |
| *SiHsf-11* | OGC12 | Null | -4392.712 | $p_{0}=\text{ 0.000},p_{1}=0.000,p_{2a}=0.589,p_{2b}=\text{ 0.411}$  Background: $\omega_{0}=0.105,\omega_{1}=\text{1.000,}\omega_{2a}=0.105,\omega_{2b}=1.000$  Foreground: $\omega_{0}=0.105,\omega_{1}=\text{1.000,}\omega_{2a}=1.000,\omega_{2b}=1.000$ | - |
|  |  | Alternative | -4392.712 | $p_{0}=0.000,p_{1}=0.000,p_{2a}=0.590,p_{2b}=0.410$  Background: $\omega_{0}=0.105,\omega_{1}=\text{1.000,}\omega_{2a}=0.105,\omega_{2b}=1.000$  Foreground: $\omega_{0}=0.105,\omega_{1}=\text{1.000,}\omega_{2a}=316.641,\omega_{2b}=316.641$ | NAN |
| *SiHsf-18* | OGC12 | Null | -3343.027 | $p_{0}=\text{ 0.000},p_{1}=0.000,p_{2a}=0.603,p_{2b}=\text{ 0.397}$  Background: $\omega_{0}=0.114,\omega_{1}=\text{1.000,}\omega_{2a}=0.114,\omega_{2b}=1.000$  Foreground: $\omega_{0}=0.114,\omega_{1}=\text{1.000,}\omega_{2a}=1.000,\omega_{2b}=1.000$ | - |
|  |  | Alternative | -3342.932 | $p_{0}=0.000,p_{1}=0.000,p_{2a}=0.604,p_{2b}=0.396$  Background: $\omega_{0}=0.114,\omega_{1}=\text{1.000,}\omega_{2a}=0.114,\omega_{2b}=1.000$  Foreground: $\omega_{0}=0.114,\omega_{1}=\text{1.000,}\omega_{2a}=270.389,\omega_{2b}=270.389$ | NAN |
| *SiHsf-09* | OGC11 | Null | -3350.169 | $p_{0}=\text{ 0.798},p_{1}=0.202,p_{2a}=0.000,p_{2b}=\text{ 0.000}$  Background: $\omega_{0}=0.086,\omega_{1}=\text{1.000,}\omega_{2a}=0.086,\omega_{2b}=1.000$  Foreground: $\omega_{0}=0.086,\omega_{1}=\text{1.000,}\omega_{2a}=1.000,\omega_{2b}=1.000$ | - |
|  |  | Alternative | -3350.169 | $p_{0}=0.798,p_{1}=0.202,p_{2a}=0.000,p_{2b}=0.000$  Background: $\omega_{0}=0.086,\omega_{1}=\text{1.000,}\omega_{2a}=0.086,\omega_{2b}=1.000$  Foreground: $\omega_{0}=0.086,\omega_{1}=\text{1.000,}\omega_{2a}=1.000,\omega_{2b}=1.000$ | NAN |
| *SiHsf-10* | OGC11 | Null | -3342.645 | $p_{0}=\text{ 0.726},p_{1}=0.166,p_{2a}=0.088,p_{2b}=\text{ 0.020}$  Background: $\omega_{0}=0.075,\omega_{1}=\text{1.000,}\omega_{2a}=0.075,\omega_{2b}=1.000$  Foreground: $\omega_{0}=0.075,\omega_{1}=\text{1.000,}\omega_{2a}=1.000,\omega_{2b}=1.000$ | - |
|  |  | Alternative | -3328.753^**^ | $p_{0}=0.743,p_{1}=0.163,p_{2a}=0.077,p_{2b}=0.017$  Background: $\omega_{0}=0.081,\omega_{1}=\text{1.000,}\omega_{2a}=0.081,\omega_{2b}=1.000$  Foreground: $\omega_{0}=0.081,\omega_{1}=\text{1.000,}\omega_{2a}=591.765,\omega_{2b}=591.765$ | 26, 6^*^, 4^**^ |
| *ZmHsf-21* | OGC16 | Null | -3647.625 | $p_{0}=\text{ 0.659},p_{1}=0.338,p_{2a}=0.002,p_{2b}=\text{ 0.001}$  Background: $\omega_{0}=0.034,\omega_{1}=\text{1.000,}\omega_{2a}=0.034,\omega_{2b}=1.000$  Foreground: $\omega_{0}=0.034,\omega_{1}=\text{1.000,}\omega_{2a}=1.000,\omega_{2b}=1.000$ | - |
|  |  | Alternative | -3647.625 | $p_{0}=0.659,p_{1}=0.338,p_{2a}=0.002,p_{2b}=0.001$  Background: $\omega_{0}=0.034,\omega_{1}=\text{1.000,}\omega_{2a}=0.034,\omega_{2b}=1.000$  Foreground: $\omega_{0}=0.034,\omega_{1}=\text{1.000,}\omega_{2a}=1.000,\omega_{2b}=1.000$ | NAN |
| *ZmHsf-09* | OGC16 | Null | -3647.054 | $p_{0}=\text{ 0.636},p_{1}=0.322,p_{2a}=0.027,p_{2b}=\text{ 0.014}$  Background: $\omega_{0}=0.033,\omega_{1}=\text{1.000,}\omega_{2a}=0.033,\omega_{2b}=1.000$  Foreground: $\omega_{0}=0.033,\omega_{1}=\text{1.000,}\omega_{2a}=1.000,\omega_{2b}=1.000$ | - |
|  |  | Alternative | -3644.361^*^ | $p_{0}=0.651,p_{1}=0.326,p_{2a}=0.025,p_{2b}=0.008$  Background: $\omega_{0}=0.034,\omega_{1}=\text{1.000,}\omega_{2a}=0.034,\omega_{2b}=1.000$  Foreground: $\omega_{0}=0.034,\omega_{1}=\text{1.000,}\omega_{2a}=998.981,\omega_{2b}=998.981$ | 3, 1^*^ |
| *ZmHsf-08* | OGC18 | Null | -2893.588 | $p_{0}=\text{ 0.784},p_{1}=0.216,p_{2a}=0.000,p_{2b}=\text{ 0.000}$  Background: $\omega_{0}=0.049,\omega_{1}=\text{1.000,}\omega_{2a}=0.049,\omega_{2b}=1.000$  Foreground: $\omega_{0}=0.049,\omega_{1}=\text{1.000,}\omega_{2a}=1.000,\omega_{2b}=1.000$ | - |
|  |  | Alternative | -2893.588 | $p_{0}=0.784,p_{1}=0.216,p_{2a}=0.000,p_{2b}=0.000$  Background: $\omega_{0}=0.049,\omega_{1}=\text{1.000,}\omega_{2a}=0.049,\omega_{2b}=1.000$  Foreground: $\omega_{0}=0.049,\omega_{1}=\text{1.000,}\omega_{2a}=1.000,\omega_{2b}=1.000$ | NAN |
| *ZmHsf-18* | OGC18 | Null | -2890.570 | $p_{0}=\text{ 0.747},p_{1}=0.199,p_{2a}=0.043,p_{2b}=\text{ 0.011}$  Background: $\omega_{0}=0.045,\omega_{1}=\text{1.000,}\omega_{2a}=0.045,\omega_{2b}=1.000$  Foreground: $\omega_{0}=0.045,\omega_{1}=\text{1.000,}\omega_{2a}=1.000,\omega_{2b}=1.000$ | - |
|  |  | Alternative | -2883.804^**^ | $p_{0}=0.769,p_{1}=0.204,p_{2a}=0.011,p_{2b}=0.006$  Background: $\omega_{0}=0.046,\omega_{1}=\text{1.000,}\omega_{2a}=0.046,\omega_{2b}=1.000$  Foreground: $\omega_{0}=0.046,\omega_{1}=\text{1.000,}\omega_{2a}=998.992,\omega_{2b}=998.992$ | 5, 2^*^ |
| *ZmHsf-11* | OGC19 | Null | -3284.694 | $p_{0}=\text{ 0.801},p_{1}=0.136,p_{2a}=0.054,p_{2b}=\text{ 0.009}$  Background: $\omega_{0}=0.090,\omega_{1}=\text{1.000,}\omega_{2a}=0.090,\omega_{2b}=1.000$  Foreground: $\omega_{0}=0.090,\omega_{1}=\text{1.000,}\omega_{2a}=1.000,\omega_{2b}=1.000$ | - |
|  |  | Alternative | -3268.299^**^ | $p_{0}=0.835,p_{1}=0.143,p_{2a}=0.019,p_{2b}=0.003$  Background: $\omega_{0}=0.090,\omega_{1}=\text{1.000,}\omega_{2a}=0.090,\omega_{2b}=1.000$  Foreground: $\omega_{0}=0.090,\omega_{1}=\text{1.000,}\omega_{2a}=999.000,\omega_{2b}=999.000$ | 7, 4^*^, 2^**^ |
| *ZmHsf-03* | OGC19 | Null | -3287.956 | $p_{0}=\text{ 0.847},p_{1}=0.153,p_{2a}=0.000,p_{2b}=\text{ 0.000}$  Background: $\omega_{0}=0.097,\omega_{1}=\text{1.000,}\omega_{2a}=0.097,\omega_{2b}=1.000$  Foreground: $\omega_{0}=0.097,\omega_{1}=\text{1.000,}\omega_{2a}=1.000,\omega_{2b}=1.000$ | - |
|  |  | Alternative | -3287.956 | $p_{0}=0.847,p_{1}=0.153,p_{2a}=0.000,p_{2b}=0.000$  Background: $\omega_{0}=0.097,\omega_{1}=\text{1.000,}\omega_{2a}=0.097,\omega_{2b}=1.000$  Foreground: $\omega_{0}=0.097,\omega_{1}=\text{1.000,}\omega_{2a}=1.000,\omega_{2b}=1.000$ | NAN |
| *OsHsf-20* | OGC22 | Null | -3475.635 | $p_{0}=\text{ 0.683},p_{1}=0.108,p_{2a}=0.180,p_{2b}=\text{ 0.028}$  Background: $\omega_{0}=0.041,\omega_{1}=\text{1.000,}\omega_{2a}=0.041,\omega_{2b}=1.000$  Foreground: $\omega_{0}=0.041,\omega_{1}=\text{1.000,}\omega_{2a}=1.000,\omega_{2b}=1.000$ | - |
|  |  | Alternative | -3475.635 | $p_{0}=0.683,p_{1}=0.108,p_{2a}=0.080,p_{2b}=0.028$ Background: $\omega_{0}=0.041,\omega_{1}=\text{1.000,}\omega_{2a}=0.041,\omega_{2b}=1.000$ Foreground: $\omega_{0}=0.041,\omega_{1}=\text{1.000,}\omega_{2a}=1.000,\omega_{2b}=1.000$ | NAN |
| *OsHsf-22* | OGC22 | Null | -3492.959 | $p_{0}=\text{ 0.842},p_{1}=0.137,p_{2a}=0.019,p_{2b}=\text{ 0.003}$ Background: $\omega_{0}=0.049,\omega_{1}=\text{1.000,}\omega_{2a}=0.049,\omega_{2b}=1.000$ Foreground: $\omega_{0}=0.049,\omega_{1}=\text{1.000,}\omega_{2a}=1.000,\omega_{2b}=1.000$ | - |
|  |  | Alternative | -3488.861^**^ | $p_{0}=0.850,p_{1}=0.137,p_{2a}=0.011,p_{2b}=0.002$ Background: $\omega_{0}=0.049,\omega_{1}=\text{1.000,}\omega_{2a}=0.049,\omega_{2b}=1.000$ Foreground: $\omega_{0}=0.049,\omega_{1}=\text{1.000,}\omega_{2a}=14.454,\omega_{2b}=14.454$ | 3, 3^*^ |
