# Supplementary material for: Adaptive evolution and divergent expression of heat stress transcription factors in grasses
Source: BMC Evol Biol. 2014 Jun 30;14:147. doi: 10.1186/1471-2148-14-147 (PMC4094458; doi:10.1186/1471-2148-14-147)
Supplement: Additional file 4 — Rice Hsf genes show statistically different expression levels in response to heat, drought, salt, and cold treatments compared to normal conditions. [file 1471-2148-14-147-S4.docx]

**Additional file 4. Rice *Hsf* genes show statistically different expression levels in response to heat, drought, salt, and cold treatments compared to normal conditions.**

| Treatment | Up/down regulation | Gene | Fold Change | q-value(%) |
| --- | --- | --- | --- | --- |
| Heat | Up | *OsHsf-08* (HsfA6) | 5.302718 | 0 |
| Heat | Up | *OsHsf-05* (HsfC2) | 10.53853 | 0 |
| Heat | Up | *OsHsf-11*(HsfA2) | 13.90098 | 0 |
| Heat | Up | *OsHsf-25* (HsfA6) | 2.147389 | 0 |
| Heat | Up | *OsHsf-01*(HsfA7) | 2.662106 | 0 |
| Heat | Up | *OsHsf-16* (HsfC2) | 3.93835 | 0 |
| Heat | Up | *OsHsf-24* (HsfB2) | 2.271979 | 0 |
| Heat | Down | *OsHsf-13* (HsfA1) | 0.371179 | 0 |
| drought | Up | *OsHsf-01*(HsfA7) | 22.49289 | 0 |
| drought | Up | *OsHsf-05* (HsfC2) | 15.27958 | 0 |
| drought | Up | *OsHsf-03* (HsfC1) | 31.42035 | 0 |
| drought | Up | *OsHsf-14* (HsfB2) | 17.88558 | 0 |
| drought | Up | *OsHsf-09* (HsfA8) | 3.436327 | 0 |
| salt | Up | *OsHsf-01*(HsfA7) | 16.88296 | 0 |
| salt | Up | *OsHsf-16* (HsfC2) | 27.23336 | 0 |
| salt | Up | *OsHsf-15* (HsfA4) | 3.637394 | 0 |
| salt | Up | *OsHsf-03* (HsfC1) | 9.396349 | 0 |
| salt | Up | *OsHsf-23* (HsfB1) | 2.967456 | 0 |
| salt | Up | *OsHsf-09* (HsfA8) | 2.166467 | 0 |
| cold | Up | *OsHsf-07* (HsfA3) | 2.886426 | 0 |
| cold | Up | *OsHsf-09* (HsfA8) | 2.611504 | 0 |
| cold | Up | *OsHsf-03* (HsfC1) | 4.914651 | 0 |
| cold | Up | *OsHsf-01*(HsfA7) | 2.027279 | 0 |
| cold | Down | *OsHsf-08* (HsfA6) | 0.307851 | 0 |
| cold | Down | *OsHsf-19* (HsfB4) | 0.490945 | 0 |
