# Supplementary material for: Adaptive evolution and divergent expression of heat stress transcription factors in grasses
Source: BMC Evol Biol. 2014 Jun 30;14:147. doi: 10.1186/1471-2148-14-147 (PMC4094458; doi:10.1186/1471-2148-14-147)
Supplement: Additional file 5 — Primer sequences used for qRT-PCR amplification. [file 1471-2148-14-147-S5.docx]

**Additional file 5. Primer sequences used for qRT-PCR amplification.**

| **Primer** | **Nucleotige sequence( 5'→ 3')** |
| --- | --- |
| *ZmHsf-01*-F | AGAGCCCAGACTTGTTCCA |
| *ZmHsf-01*-R | ACCGCCCTTCATCCCT |
| *ZmHsf-02*-F | AAACTTCCAATCAGCCAATA |
| *ZmHsf-02*-R | TGTCATCCCATTCACATAGA |
| *ZmHsf-03*-F | GCCGTTCCTCACCAAGA |
| *ZmHsf-03*-R | CGGTGTATGTCGCAGAGC |
| *ZmHsf-04*-F | TCTTGCCTGGAGGTTGG |
| *ZmHsf-04*-R | GGTTCCGCATTGCTCTT |
| *ZmHsf-05*-F | GATGACCCAACCACGAA |
| *ZmHsf-05*-R | CGAGGTAAGAGCCAAGAG |
| *ZmHsf-06*-F | AAGCGTCTTCAAGGGATG |
| *ZmHsf-06*-R | CACTGGATTTCAGGAGGG |
| *ZmHsf-07*-F | CACTTCCCGCTCTTCCA |
| *ZmHsf-07*-R | CGGCTCCCTCCAACAAT |
| *ZmHsf-08*-F | GAGCAAGACGCACCAGAT |
| *ZmHsf-08*-R | CGGACAAAGGAGGAGAAGT |
| *ZmHsf-09*-F | TGCGGCGTTGTTGG |
| *ZmHsf-09*-R | GCCTCCTGCTTCTCGTC |
| *ZmHsf-10*-F | GTCCAAGCCTCAGGTAGAAG |
| *ZmHsf-10*-R | CTCCCTGGCGTAGTTGC |
| *ZmHsf-11*-F | GGGCGGAAACGAATG |
| *ZmHsf-11*-R | TGGGCCGGTAAATGG |
| *ZmHsf-12*-F | ACGGGTTTAGAAAGGTTGA |
| *ZmHsf-12*-R | TGCTGTGGTTGGCTGTTA |
| *ZmHsf-13*-F | CGTACCGCATGGTGGA |
| *ZmHsf-13*-R | AAGCCGTAGGTGTTGAGC |
| *ZmHsf-14*-F | TGAGCGTGCGTTATTTGA |
| *ZmHsf-14*-R | GCCATTGCAGGACAGAGT |
| *ZmHsf-15*-F | CTGACAGATGGGAGTTTGC |
| *ZmHsf-15*-R | CAGATTCCAGCCTGTGATT |
| *ZmHsf-16*-F | CTCCCTGTGCGACATCC |
| *ZmHsf-16*-R | GGCTCGGCATCCAAAA |
| *ZmHsf-17*-F | GCTCAGAATGCCACAAACC |
| *ZmHsf-17*-R | CTGCCTTAGCTTCACCACTT |
| *ZmHsf-18*-F | TGACCAAGACGCACCAGAT |
| *ZmHsf-18*-R | GCGGACGAAGGAGGAGA |
| *ZmHsf-19*-F | CTTCTCCAGCTTTGTGCG |
| *ZmHsf-19*-R | CCTTCCGTCGGTGTATGT |
| *ZmHsf-20*-F | GACCTCAGGGCAAGACCA |
| *ZmHsf-20*-R | AACCGCTCCCAGAATACA |
| *ZmHsf-21*-F | GGGCAGCGAGAACAACA |
| *ZmHsf-21*-R | GCCACGCAGGAACGACT |
| *ZmHsf-22*-F | GCGGCAACTGAACACCTA |
| *ZmHsf-22*-R | CCTCCTCATAATCCCTCCT |
| *ZmHsf-23*-F | GGACAGGTGGGAGTTCG |
| *ZmHsf-23*-R | AACGGCTGGGATGGAA |
| *ZmHsf-24*-F | CGCCAGCTCAACACCTAC |
| *ZmHsf-24*-R | CCCATCATCATTCCACCA |
| *ZmHsf-25*-F | CTTCTTCCCAAGCACTTCA |
| *ZmHsf-25*-R | CAAACTCCCATCTGTCCC |
| *ZmHsf-26*-F | TCAACACCTATGGCTTCCG |
| *ZmHsf-26*-R | GGCGTCCTTCCTCTTTCC |
| *ZmHsf-27*-F | GGCAGACAGGTGGGAGT |
| *ZmHsf-27*-R | GAGGCTGAGGTCGTAAGG |
| *ZmHsf-28*-F | GGAAGATAGTGGCGGACAG |
| *ZmHsf-28*-R | GGAGAACGGGCTGAGGT |
| *ZmActin1*-F | GATGATGCGCCAAGAGCTG |
| *ZmActin1*-R | GCCTCATCACCTACGTAGGCAT |

Note: The maizeGDB locus of gene *ZmActin1* is GRMZM2G126010.
